# Effect of Interferon-*γ* on the Basal and the TNF*α*-Stimulated Secretion of CXCL8 in Thyroid Cancer Cell Lines Bearing Either the RET/PTC Rearrangement Or the BRAF V600e Mutation

**DOI:** 10.1155/2016/8512417

**Published:** 2016-07-31

**Authors:** Mario Rotondi, Francesca Coperchini, Oriana Awwad, Patrizia Pignatti, Christian A. Di Buduo, Vittorio Abbonante, Flavia Magri, Alessandra Balduini, Luca Chiovato

**Affiliations:** ^1^Unit of Internal Medicine and Endocrinology, Fondazione Salvatore Maugeri I.R.C.C.S., Laboratory for Endocrine Disruptors and Chair of Endocrinology University of Pavia, 27100 Pavia, Italy; ^2^Department of Biopharmaceutics and Clinical Pharmacy, The University of Jordan, Amman 11937, Jordan; ^3^Allergy and Immunology Unit, Fondazione Salvatore Maugeri I.R.C.C.S., 27100 Pavia, Italy; ^4^Department of Molecular Medicine, University of Pavia, 27100 Pavia, Italy; ^5^Biotechnology Research Laboratories, I.R.C.C.S., Policlinico San Matteo Foundation, 27100 Pavia, Italy; ^6^Department of Biomedical Engineering, Tufts University, Medford, MA 02155, USA

## Abstract

CXCL8 displays several tumor-promoting effects. Targeting and/or lowering CXCL8 concentrations within the tumor microenvironment would produce a therapeutic benefit. Aim of this study was to test the effect of IFN*γ* on the basal and TNF*α*-stimulated secretion of CXCL8 in TCP-1 and BCPAP thyroid cancer cell lines (harboring RET/PTC rearrangement and BRAF V600e mutation, resp.). Cells were incubated with IFN*γ* (1, 10, 100, and 1000 U/mL) alone or in combination with TNF-*α* (10 ng/mL) for 24 hours. CXCL8 and CXCL10 concentrations were measured in the cell supernatants. IFN*γ* inhibited in a dose-dependent and significant manner both the basal (ANOVA* F*: 22.759; *p* < 0.00001) and the TNF*α*-stimulated (ANOVA* F*: 15.309; *p* < 0.00001) CXCL8 secretions in BCPAP but not in TPC-1 cells (NS). On the other hand, IFN*γ* and IFN*γ* + TNF-*α* induced a significant secretion of CXCL10 in both BCPAP (*p* < 0.05) and TPC-1 (*p* < 0.05) cells. Transwell migration assay showed that (i) CXCL8 increased cell migration in both TPC-1 and BCPAP cells; (ii) IFN*γ* significantly reduced the migration only of BCPAP cells; and (iii) CXCL8 reverted the effect of IFN*γ*. These results constitute the first demonstration that IFN*γ* inhibits CXCL8 secretion and in turn the migration of a BRAF V600e mutated thyroid cell line.

## 1. Introduction

CXCL8 is a key regulator of immune cells infiltration into the tumor microenvironment promoting cancer invasion and metastasis [1, 2]. CXCL8 is detected in serum and tissue specimens from patients with solid tumors, its circulating levels being correlated with tumor size, depth of infiltration, stage, and prognosis [3]. Lower serum levels of CXCL8 characterize a less aggressive course of malignancy and a better response to anticancer therapy [3–5]. These data support the concept that lowering the levels of CXCL8 in the tumor microenvironment would be of benefit in cancer patients [3, 6, 7]. The role of CXCL8 in tumor progression [8, 9] and the therapeutic benefits derived from targeting/lowering this chemokine were also demonstrated in thyroid cancer [10, 11].* In vivo* experiments showed that treatment with an anti-CXCL8 neutralizing antibody abrogated the invasiveness of papillary thyroid cancer cells in mice transplanted with a human thyroid cancer cell line [11]. In parallel to CXCL8 targeting experiments, several pharmacological compounds were tested for their ability to inhibit the secretion of this chemokine [10, 12]. However, the inhibition of CXCL8 secretion turned out to be rather complicated due to the multiple intracellular signals and/or pathways that mediate its release [1, 13]. It is known that CXCL8 is primarily regulated by NF-*κ*B and/or activator protein-1 (AP1) mediated transcriptional activity. CXCL8 secretion also results from inflammatory signals (e.g., tumor necrosis factor *α*, IL-1*β*), which regulate CXCL8 expression [1]. With specific regard to cancer, the activation of several oncogenes further modulates the release of CXCL8 [13–15].

It was recently reported that metformin, a drug with a wide spectrum of anticancer effects [16–18], significantly inhibits the secretion of CXCL8 induced by TNF*α* in primary cultures of human thyroid cells, derived both from the normal gland parenchyma and from surgical specimens of papillary thyroid cancer with unknown genetic background. At variance with these results, metformin did not affect the TNF-*α*-stimulated secretion of CXCL8 in thyroid cancer cell lines harboring the RET/PTC rearrangement or the BRAF V600e mutation (TPC-1 and BCPAP cells, resp.) [12]. Type I and type II interferons (IFNs) also reduce the secretion of CXCL8 in cultured human cells originated from several organs including the thyroid [19–21]. Among IFNs, IFN*γ* was identified as the most powerful inhibitor [21]; however, its ability to reduce the secretion of CXCL8 in thyroid cancer cells was not investigated previously.

Aim of the present study was thus to investigate whether IFN*γ* inhibits the basal and the TNF-*α*-stimulated secretion of CXCL8 in TCP-1 and BCPAP thyroid cancer cells.

## 2. Materials and Methods

### 2.1. Thyroid Tumor Cell Lines

Human thyroid cancer cell lines, TPC-1 and BCPAP (harboring the RET-PTC rearrangement and the BRAF V600e mutation, resp.) were a kind gift from Professor M. Santoro (Medical School, University “Federico II” of Naples, Naples, Italy). All cell lines had been previously tested and authenticated by DNA analysis to be of thyroid origin. Cancer cells were propagated in Dulbecco's Modified Eagle Medium (DMEM) (Sigma, Saint Louis, MO, USA) supplemented with 10% fetal bovine serum (Sigma, Saint Louis, MO, USA), 2 mM L-glutamine, and 100 U/mL penicillin-streptomycin (Sigma, Saint Louis, MO, USA).

### 2.2. Basal and TNF-*α*-Stimulated Secretion of CXCL8 and CXCL10 in TPC-1 or BCPAP Cells in the Presence or Absence of Increasing Concentrations of IFN*γ*

For the CXCL8 secretion assays, 3000 cells were seeded into 96-well plates in complete medium. After adherence to the plastic surface, cells were incubated for 24 h in serum-free medium (basal condition) with or without increasing concentrations of IFN*γ* (1, 10, 100, and 1000 U/mL) (R&D systems, Minneapolis). In a second set of experiments, TPC-1 and BCPAP cells were treated with TNF-*α* 10 ng/mL (stimulated condition) alone or in combination with the same concentrations of IFN*γ*. All experiments were performed in triplicate.

To test the responsiveness of TPC-1 and BCPAP cells to IFN*γ*, the levels of CXCL10 (a prototype interferon-*γ*-inducible chemokine) [22–24] were measured in the supernatants (the same used for the assay of CXCL8) of thyroid cancer cell lines after treatment with TNF*α* 10 ng/mL and IFN*γ* 1000 U/mL alone or in combination.

### 2.3. ELISA for Chemokines

The concentrations of CXCL8 and CXCL10 in thyroid cell supernatants were measured using commercially available kits (R&D Systems, Minneapolis). The mean minimum detectable dose of CXCL8 was 3.5 pg/mL. The intra- and interassay coefficients of variation were 3.4 and 6.8%, respectively. The mean minimum detectable dose of CXCL10 was 1.67 pg/mL. The intra- and interassay coefficients of variation were 3.0 and 6.9%, respectively. Samples were assayed in duplicate. Quality control pools of low, normal, or high concentrations were included in each assay.

### 2.4. Cell Migration Assay

The cell migration assay was performed with the transwell migration chamber system (Merck Millipore, Milan, Italy), as previously described [25]. Briefly, BCPAP and TPC-1 were cultured for 24 hours with fresh medium alone or supplemented with 1000 U/mL of IFN*γ*, 50 ng/mL of CXCL8 (R&D Systems, Minneapolis), or their combination. After treatment, 20 × 10^3^ cells/well were seeded in the upper chambers of the 96-well plate with polycarbonate inserts having 0.3 cm^2^/well membrane area and 8 *μ*m pore size. In each condition, the lower chambers were filled with 150 *μ*L of the corresponding medium. The cells were left to migrate for 16 hours at 37°C and 5% CO_2_. At the end of the incubation, samples were analyzed as previously described [26]. Briefly, cell inserts were washed three times with PBS and migrated cells on the underside of the membrane were fixed with 4% paraformaldehyde for 20 minutes. Cell nuclei were then stained with Hoechst 33258 (1 : 10000) (Life Technologies, Monza, Italy). Finally, the membranes were cut out with a scalpel and mounted onto glass slides with ProLong Gold antifade reagent (Life Technologies, Monza, Italy). Three replicates have been performed for each condition. Images were acquired using an Olympus BX51 microscope (Olympus, Deutschland GmbH, Hamburg, Germany). The number of migrated cells was blind-counted analyzing 12 random fields of the membranes per condition. Data are expressed as means of number of migrated cells ± standard deviation (SD).

### 2.5. Wound-Healing Assay

For wound-healing assay cells were seeded in a 24-well plate. When cells reached nearly the 90% of cell confluence, a scratch wound was created in the monolayer using a sterile 20 *μ*L pipette tip. Cells were then treated with fresh medium alone or supplemented with 1000 U/mL of IFN*γ* (R&D Systems, Minneapolis). Phase contrast images were captured between 0 and 24 hours using an Olympus IX53 microscope (Olympus, Deutschland GmbH, Hamburg, Germany). Data are expressed as the percentages of the remaining gap area after 24 hours relative to the initial gap area (0 hours). The area was measured using the LCmicro software (Olympus Soft Imaging Solutions GmbH).

### 2.6. Statistical Analysis

Statistical analysis was performed using SPSS software (SPSS, Inc., Evanston, IL). Mean group values were compared through one-way ANOVA test for normally distributed variables.* Post hoc* analysis was performed applying Bonferroni's correction. The different effect of IFN*γ* on BCPAP cells in basal condition and after stimulation with TNF*α* was assessed by ANOVA for repeated measures for all the concentrations of IFN*γ*. Between-group comparisons were performed by Student's *t*-test for independent samples. Values are reported as mean ± SD unless otherwise noted. A *p* value < 0.05 was considered statistically significant.

## 3. Results

### 3.1. Effect of IFN*γ* on CXCL8 Secretion in BCPAP and TPC-1 Cells

**Figure 1:**
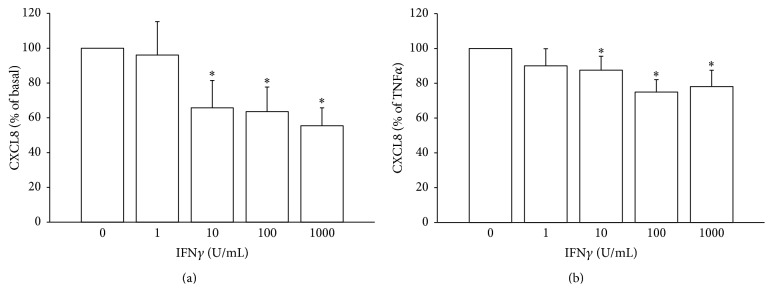
IFN*γ* inhibits the secretion of CXCL8 in BCPAP cells. (a) IFN*γ* significantly and dose-dependently inhibits the basal CXCL8 secretion (ANOVA* F*: 22.75; *p* < 0.00001;* post hoc* analysis by Bonferroni: ^*∗*^ *p* < 0.001 versus basal). (b) IFN*γ* significantly inhibits the TNF*α*-stimulated CXCL8 secretion (ANOVA* F*: 15.30; *p* < 0.00001;* post hoc* analysis by Bonferroni: ^*∗*^ *p* < 0.001  and  °*p* < 0.01 versus TNF*α*). The inhibitory effect of IFN*γ* is expressed as percentage of basal and TNF*α*-induced levels of CXCL8, which were estimated as 100%.

CXCL8 was measured in the basal and TNF-*α*-stimulated condition in BCPAP and in TPC-1 cells. As previously reported [27], the incubation with TNF-*α* elicited a significant increase of CXCL8 concentration in the supernatants of both cell lines. Treatment with IFN*γ* produced a significant and dose-dependent inhibition of both the basal (ANOVA* F*: 22.759; *p* < 0.00001) and the TNF-*α*-stimulated (ANOVA* F*: 15.309; *p* < 0.00001) secretion of CXCL8 in BCPAP cells. In order to compare the magnitude of inhibition of CXCL8 secretion between the basal and the stimulated condition (TNF*α*), ANOVA for repeated measures was performed. Results showed that the inhibiting effect of IFN*γ* on CXCL8 secretion was significantly greater in the basal as compared with the TNF-*α*-stimulated condition (ANOVA* F*: 10.673; *p* < 0.005) (Figures 1(a) and 1(b)).

**Figure 2:**
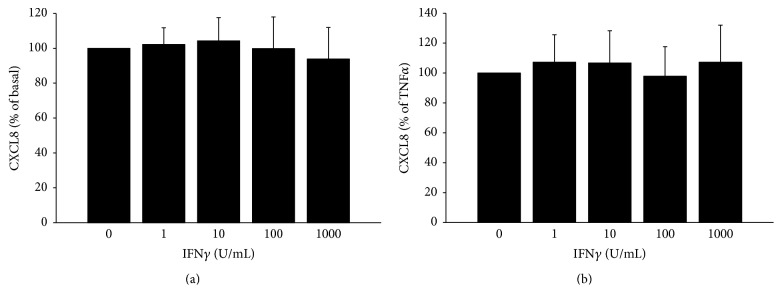
IFN*γ* does not inhibit either the basal (ANOVA* F*: 0.985; *p* = 0.423) (a) or the TNF*α*-stimulated (ANOVA* F*: 0.685; *p* = 0.606) (b) CXCL8 secretion in TPC-1 cells. The inhibitory effect of IFN*γ* is expressed as percentage of basal and TNF*α*-induced levels of CXCL8 which were estimated as 100%.

On the other hand, IFN*γ* had no inhibitory effect on the secretion of CXCL8 in TPC-1 cells, either in basal (Figure 2(a)) or in TNF*α*-stimulated condition (Figure 2(b)) (NS for both conditions).

### 3.2. Effect of IFN*γ* on CXCL10 Secretion in BCPAP and TPC-1 Cells

To rule out that TPC1 cells (in which IFN*γ* did not reduce CXCL8 secretion) were refractory to IFN*γ*, the interferon-*γ*-inducible chemokine (CXCL10) was also measured.

**Figure 3:**
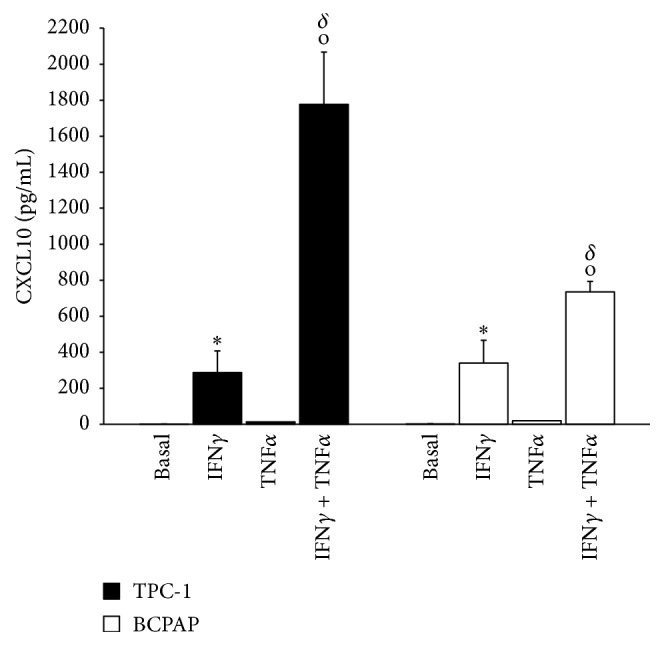
CXCL10 secretion in TPC-1 and BCPAP cells in basal condition, after treatment with IFN*γ* 1000 U/mL and TNF*α* 10 ng/mL alone or in combination. ^*∗*^ *p* < 0.05 versus basal; °*p* < 0.05 versus IFN-*γ*; ^*δ*^ *p* < 0.05 TPC-1 versus BCPAP.

CXCL10 was virtually absent in the supernatants of both BCPAP and TPC-1 cells in basal condition. IFN*γ* significantly increased CXCL10 secretion in both BCPAP (339.1 ± 127.6 pg/mL; *p* < 0.05 versus basal) and TPC-1 (286.5 ± 120.8 pg/mL; *p* < 0.05 versus basal) cells, whereas TNF*α* alone had no detectable effect. However, the combined stimulation with IFN*γ* + TNF*α* produced a significant synergistic effect on CXCL10 secretion in both BCPAP (735.7 ± 57.5 pg/mL; *p* < 0.05 versus IFN*γ*) and TPC-1 (1776.5 ± 291.2; *p* < 0.05 versus IFN*γ*) being higher in TPC-1 cells (TPC-1 versus BCPAP cells  *p* < 0.05) (Figure 3).

### 3.3. Migration Assays

The above results demonstrated the ability of IFN*γ* to reduce CXCL8 secretion in BCPAP cells and deserved to be confirmed in terms of ability to interfere with any of the CXCL8 mediated tumor-promoting activities. In view of the well established role of CXCL8 in promoting the metastatic spread of tumors, cell migration assays were performed.

**Figure 4:**
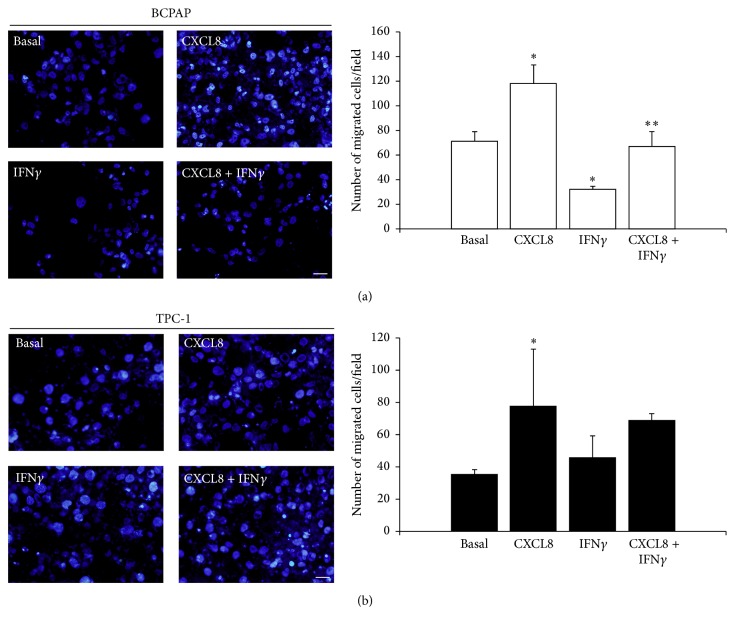
Representative images and the respective histograms of (a) BCPAP and (b) TPC-1 after 16 hours of migration within the transwell migration chamber system in different conditions. Nuclei were counterstained with Hoechst 33258 in blue (images were acquired by an Olympus BX51, magnification 20x, scale bar = 50 *μ*m). Bar graphs show the corresponding analysis of migrated cells on the lower side of the transwell filter. BCPAP: ANOVA* F* = 58.71; *p* < 0.0001;* post hoc* analysis by Bonferroni: *p* < 0.001 CXCL8 versus basal; *p* < 0.05 IFN*γ* versus basal; *p* < 0.005 IFN*γ* + CXCL8 versus IFN*γ*. TPC-1: ANOVA* F* = 8.85 *p* < 0.05;* post hoc* analysis by Bonferroni: *p* < 0.05 CXCL8 versus basal; NS IFN*γ* versus basal; NS IFN*γ* + CXCL8 versus IFN*γ*. ^*∗*^ *p* < 0.05 versus basal; ^*∗∗*^ *p* < 0.05 versus CXCL8 + IFN*γ*.

The results of the transwell migration assay are showed in Figure 4. Briefly, the mean of the number of migrated cells per field observed in basal condition was clearly increased by treatment with CXCL8 both for BCPAP (basal: 71.4 ± 7.6; CXCL8: 118.22 ± 10.9) and TPC-1 (basal: 35.8 ± 2.5; CXCL8: 77.7 ± 18.1). Of note, treatment with IFN*γ* reduced the basal migration of BCPAP cells while the combined treatment with IFN*γ* + CXCL8 reverted the effect of IFN*γ* (basal: 71.4 ± 7.6; IFN*γ*  32 ± 1.7; IFN*γ* + CXCL8: 67 ± 8.7). On the contrary, IFN*γ* did not significantly influence the migration ability of TPC-1 cells.

**Figure 5:**
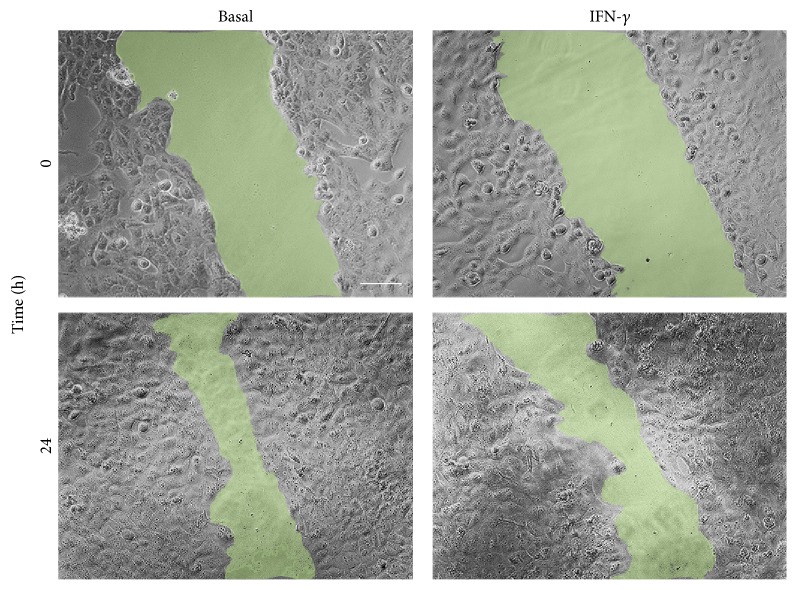
Wound-healing assay for BCPAP cells in basal condition (time 0: 0%; after 24 h: 51.1% of wound closure) and after treatment with IFN*γ* (time 0: 0%; 24 h: 38.3% of wound closure).

The effect of IFN*γ* on the migration of the BCPAP cells was corroborated by the wound-healing assay. As shown in Figure 5, the treatment with IFN*γ*, at least in part, inhibited the wound closure further confirming its ability to reduce the BCPAP cells migration.

## 4. Discussion

The present study demonstrates that IFN*γ* inhibits the basal and TNF*α*-stimulated secretion of CXCL8 in BCPAP, but not in TPC-1 thyroid cancer cell lines. This is the first evidence showing that IFN*γ* inhibits CXCL8 secretion in a BRAF V600e mutated cancer cell line.

Previous attempts aimed at inhibiting CXCL8 secretion with metformin were successful in primary cultures of human normal thyroid cells and in cultures prepared from surgical specimens of papillary thyroid cancer. However, no inhibitory effect was elicited in thyroid cancer cell lines [12].

This different ability of metformin to inhibit the secretion of CXCL8 suggested that in normal thyrocytes and thyroid cancer cell lines bear different secretory pathways of CXCL8 which would imply that specific inhibitory strategies are required [12, 27].

This concept is further supported by the here reported results which show that IFN*γ* inhibits the secretion of CXCL8 in BRAF V600e mutated BCPAP cells, but not in TPC-1 cells bearing a RET/PTC rearrangement.

The present study was specifically designed to test the possibility to inhibit the secretion of CXCL8 in thyroid cancer cells harboring specific genetic mutations and the characterization of the IFN*γ* signaling producing this inhibition appears by far behind the aim of our study. The lack of mechanistic experiments, which we acknowledge as a limitation of the study, would prevent drawing conclusions on the mechanisms by which IFN*γ* exerts this inhibitory effect on CXCL8. However, the similar response of BCPAP and TPC-1 to IFN*γ* in terms of induction of CXCL10 secretion demonstrates that functional IFN*γ* receptors are expressed at similar levels on the cell surface of both cell lines and fits with the notion that IFNs specifically inhibit the expression of CXCL8, while they activate other NF-kB regulated genes [20].

CXCL10 is a chemokine which is basally not secreted by thyrocytes, it is strongly upregulated by IFN*γ*. TNF*α* does not induce CXCL10 secretion but it strongly synergizes with IFN*γ*. The mechanism of this synergism is due to the fact that TNF*α* up-regulates at both mRNA and protein as well as increases the membrane expression of IFN*γ* receptors [28, 29]. Thus, the finding that following stimulation with IFN*γ* + TNF*α*, a higher level of CXCL10 was secreted by TPC-1 as compared to BCPAP indicates that TNF*α* induced a higher upregulation of IFN*γ* receptors in TPC-1. This finding is in line with a recent evidence provided by our group that TPC-1 cells are more sensitive to TNF*α* than BCPAP and it would further sustain the concept that multiple pathways are involved in CXCL8 secretion, thus it is not surprising that inhibitory agents may also be different.

In other words, the explanation could be that IFN*γ* stimulates CXCL10 secretion by acting on a mechanism shared by both cell types, while it would inhibit the release of CXCL8 by modulating a specific pathway, which is active only in BCPAP cells. Taken together the above considerations further support the concept that the presence of a specific genetic lesion would “switch on” different mechanisms/pathways involved in CXCL8 secretion.

In addition, the CXCL8-inhibitory effect of IFN*γ* in BCPAP cells appears significantly stronger on the basal as compared with the TNF*α*-stimulated secretion. Again, this differential behavior suggests that the secretion of CXCL8 is regulated through complex mechanisms, which involve multiple intracellular signals and/or pathways [1, 30, 31]. These are profoundly different according to cell-related (the specific genetic lesion of cancer cells) and condition-related (basal and stimulated secretion of CXCL8 in cells with the same genetic mutation) aspects.

The finding that IFN*γ* inhibits the secretion of CXCL8 in BCPAP cells prompted us to evaluate whether cell migration, a well characterized CXCL8 mediated tumor-promoting activity [9, 11], was also affected by IFN*γ* treatment. Transwell migration assays confirmed that CXCL8 promotes cell migration both in TPC-1 and in BCPAP which appears in line with the recent findings provided by two independent studies [9, 11].

Furthermore, while IFN*γ* did not affect the number of migrated TPC-1 cells, it reduced the number of migrated BCPAP cells, this effect being promptly reverted by addition of CXCL8 to IFN*γ*. The results obtained by wound-healing assays further confirmed the ability of IFN*γ* to inhibit the migration of BCPAP cells.

The fact that CXCL8 increased migration of both TPC-1 and BCPAP cells together with the fact that treatment with IFN*γ* produced a reduction in the number of migrated cells only in BCPAP (in which IFN*γ* significantly lowered the secretion of CXCL8) constitutes a further confirmation of the tumor-promoting effect of CXCL8 and of the need to find novel and more effective inhibitory strategies.

In conclusion, the results of the present study indicate that IFN*γ*, among its pleiotropic effects, is also able to inhibit CXCL8 secretion and cell migration in BRAF V600e mutated BCPAP but not in TPC-1 thyroid cancer cell line bearing the RET/PTC rearrangement. Given the advantages of lowering this chemokine levels in the tumor microenvironment further efforts targeting CXCL8 inhibition appear worthwhile.
